# From SMILES Codes for Reactants and Products to Transition States With VeloxChem

**DOI:** 10.1002/jcc.70454

**Published:** 2026-07-03

**Authors:** Bastiaan van Hoorn, Patrick Norman, Mårten S. G. Ahlquist

**Affiliations:** ^1^ Division of Theoretical Chemistry and Biology School of Engineering Sciences in Chemistry, Biotechnology and Health, KTH Royal Institute of Technology Stockholm Sweden

## Abstract

A force field interpolation method for generating initial guesses for transition state optimizations is presented. The user supplies reactants and products as SMILES strings (or *XYZ* coordinates), after which automatically constructed force fields are interpolated to approximate the potential energy surface (PES) along the reaction coordinate. The transition state guess is obtained by sampling along this approximate PES. Reliance on force fields ensures the method is transparent in its workings and computationally inexpensive. The workflow is implemented in VeloxChem, enabling Jupyter notebook execution with a few lines of Python code. An interactive widget makes visualization and inspection of results easy and intuitive, and a flexible Python API facilitates integration into complex automated workflows. The method is demonstrated on ten chemically diverse systems, including a transition‐metal catalytic cycle. Benchmarking across 121 reactions resulted in convergence for 115 transition states, with an average cost of 107 gradient evaluations per reaction—comparable to more demanding double‐ended methods.

## Graphical abstract

Initial guesses for transition state optimizations are produced from a mere input of reactant and product SMILES codes, and achieved by interpolating generated force fields for reactants and products. The entire workflow scheme is implemented in the VeloxChem software.

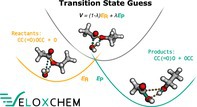

## 1 Introduction

The accurate identification of transition states (TSs) is a cornerstone of theoretical chemistry, providing the foundation for understanding reaction mechanisms and predicting kinetic behavior. Within the framework of transition state theory (TST) and its harmonic formulation, reaction rates are governed by the free‐energy barrier separating reactants and products on the potential energy surface (PES) [1, 2]. The maximum of this barrier corresponds to a first‐order saddle point, defined by a single imaginary vibrational frequency along the reaction coordinate. Because the rate constant depends exponentially on the barrier height, even modest errors in the computed energy can lead to substantial deviations in predicted kinetics. Precisely locating this saddle point is therefore essential for accurately describing elementary reaction steps and constructing reliable kinetic models [3]. However, locating and optimizing the TS is inherently a nontrivial task. Algorithms must track the uphill potential along the reaction coordinate while simultaneously minimizing in all other directions. The possible presence of multiple competing pathways with near‐degenerate saddle points, as well as flat or bifurcating regions of the PES, further complicate the search.

These difficulties have motivated the development of a wide range of TS optimisation methods over the years. Single‐ended methods, such as eigenvector‐following [4, 5, 6] and the dimer method [7, 8], start from a single molecular structure and attempt to drive the optimization toward the nearby saddle point. However, their success hinges on the availability of an initial geometry that lies sufficiently close to the true TS. For obtaining such an initial guess, traditional workflows rely on constrained optimisation scans or geometric interpolations between reactant and product structures [9], while more recent efforts incorporate chemical topology [10], mechanistic templates [11], or machine‐learning‐based predictors to generate more reliable starting points [12]. However, application of single‐ended methods for large scale automated exploration of reaction mechanisms remains limited by the inherent need for user guidance.

Double‐ended methods adopt a conceptually distinct strategy by starting from two known minima on the potential energy surface and constructing the minimum‐energy pathway (MEP) that connects them. The TS is then located at the point where the energy is maximized along this pathway [13]. Examples of such methods include the nudged‐elastic‐band (NEB) method [14], the quadratic string method [15], and the freezing string method [16]. Because these approaches generate information about the entire reaction path and do not require an explicit TS guess, they are better suited to automated exploration of complex reactions. By breaking off these methods before full convergence, structures along the path can be used as initial guesses for single‐ended TS optimizations, improving performance [17, 18]. However, the computational cost of these methods remains significantly higher than that of single‐ended searches, as multiple images along the reaction path must simultaneously be optimized, restricting the use of these methods to small systems.

Although classical force fields are typically not designed to capture the quantum mechanical (QM) nature of bond breaking and formation, their computational efficiency has motivated the development of specialized molecular mechanics (MM) methods for this purpose. Strategies to bridge this gap include bond‐order dependent potentials such as ReaxFF [19], as well as potentials derived directly from QM data like QMDFF [20]. Despite their utility in specific contexts, these methods share persistent limitations: parameter optimization is demanding, often requiring system‐specific empirical tuning, and the resulting models transfer poorly to new chemical environments [21]. The advent of machine‐learning force fields has offered a more flexible alternative, with recent work extending their applicability to reactive [22] and excited‐state dynamics [23, 24]. Nevertheless, these models remain fundamentally bounded by the diversity of their training data, and generalization to previously unseen chemical systems remains an open challenge.

Alternative approaches include hybrid QM/MM strategies, which embed small‐scale quantum calculations within a larger classical environment [25, 26]. QM/MM methods have been successfully used to investigate reactivity in diverse large heterogeneous systems. However, large‐scale automation remains challenging because the key methodological considerations are in general system dependent, most notably system partitioning, QM/MM boundary treatment, and charge interaction between the QM and MM region [27]. Related to this are the empirical valence bond (EVB) method [28], which uses established classical fields to sample reaction pathways, and the Q2MM method [25], which uses data from optimized TSs to create force fields to sample TSs. However, since these methods rely on fitting to pre‐calculated QM data, the generation of this data remains a bottleneck.

The methods described above have increasingly been integrated into sophisticated automated workflows. In these frameworks, classical or semi‐empirical techniques are combined with structural templates to generate initial guesses that serve as starting points for higher‐level quantum chemical methods and possible generation of new templates. Such hierarchical strategies enable robust iteration over multi‐step mechanisms and, in some cases, exhaustive exploration of entire reaction spaces under controlled computational protocols [29, 30, 31]. However, because the tools used in these workflows are often tightly interconnected, integrating them with other software environments remains difficult. More recently, machine‐learning‐driven approaches have been introduced to accelerate or substitute individual stages of TS discovery. These efforts include models designed to propose reactive geometries, guide search directions, or directly generate TS structures [32, 33, 34]. Nonetheless, requirement of sufficient amounts of training data as well ensuring reliable transferability of two new reaction types are still limiting factors.

In this work we present a method that uses interpolated force fields inspired by EVB to generate initial TS guesses with minimal computational requirements. By performing optimizations of the structure at various interpolations of a reactant and product force field, a relative MM energy is obtained. The structure corresponding to the highest relative energy serves as a remarkably reliable starting guess for a single‐ended TS optimization. A more accurate guess can be obtained by performing a conformational sampling at various interpolations, or by calculating the energy with any QM method of choice and picking the structure corresponding to the highest QM energy instead. This approach provides a robust, flexible, and computationally inexpensive way to generate TS guesses for a wide diversity of systems.

This workflow has been implemented in the open source VeloxChem [35] program and can be executed with just a few lines of Python code on a personal computer. The input can be given as SMILES strings, and the results can be visualized with an interactive widget in a Jupyter notebook. This tool will be helpful for experienced computational chemists dealing with complex systems, but equally it can be instructive for students taking their first quantum chemistry course. All the while, an easily accessible Python interface enables flexible automation and modification for more advanced users. To demonstrate the method, it has been applied to a range of representative systems of various sizes up to 250 atoms. Furthermore, to benchmark the performance of the proposed method, it has been applied to 121 reactions from a slightly modified version of the set of reactions used to benchmark the NEB‐TS method in Ref. [18].

In the following section, the theoretical framework of the method is outlined. An overview of the reaction mapping algorithm is provided, followed by a detailed description of the interpolated force field construction and sampling. Subsequently, a series of results from larger demonstrative systems are presented to illustrate the applicability of the proposed method. Finally, the results obtained from a benchmark set are analyzed and discussed.

## 2 Methodology

**FIGURE 1:**
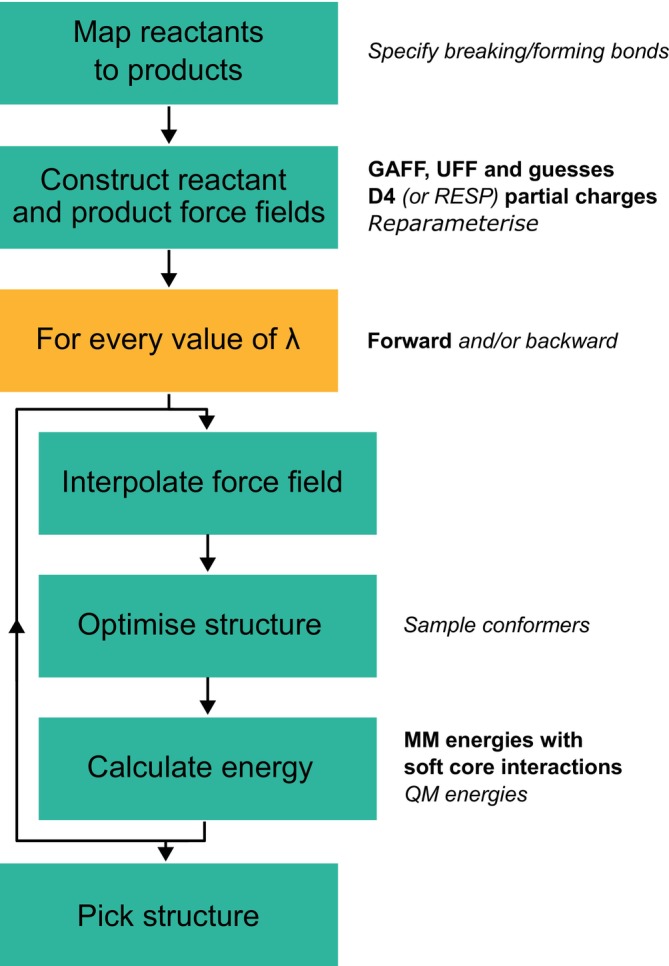
Schematic overview of the workflow. Bold‐faced text indicates default settings. Italic text indicates optional settings.

This section provides a detailed description of the workflow steps, as depicted in Figure 1. It begins by addressing the reaction mapping problem and the solution employed. Next, the construction and interpolation of the force field are explained. Finally, the section covers the sampling and energy calculation procedures applied to obtain the transition state guess.

### 2.1 Reaction Mapping

In order to correctly interpolate between the reactant and product force fields, it is crucial to know which atom in the reactant corresponds to which atom in the product. This is known as the atom‐atom‐mapping or reaction mapping problem. While this problem is relatively straightforward to address for a chemist, it is difficult to implement algorithmically. Formally, the space of possible solutions scales factorially with the number of atoms of a given element, and even verifying a given solution requires comparison to a factorially growing subset of these solutions [36]. In most approaches, the problem is cast in terms of graph theory, representing molecular structures as graphs with atoms as nodes and bonds as edges. The problem then becomes to find the bijection between the nodes of the reactant graph R and product graph P such that the element of every atom is kept constant while minimizing the number of edges that are broken or formed, along with other possible chemical constraints such as bond‐order preservation and energy minimization.

The graph isomorphism problem involves finding an (isomorphic) mapping between two graphs of the same size and connectivity, and efficient heuristic algorithms such as VF2 [37, 38] and VF2++ [39] have been developed to address it. Because of this, once one has identified the forming and breaking bonds in a reaction, the entire reaction mapping is comparatively easy to perform. The subgraph isomorphism problem involves finding a mapping between a smaller and larger graph such that the smaller graph fits in the larger graph while preserving connectivity. One common approach to the reaction mapping problem is to find the maximum overlapping substructure, known as the maximal common subgraph approach [40], which has been improved upon with various weighting schemes [41, 42]. Other approaches include iterating in various ways over all possible combinations of bond breaking and forming [43] and optimization based techniques [44, 45]. The approach implemented for this work combines a maximal common subgraph approach with bond‐breaking iteration.

Consider a reactant graph R and product graph P, where R has at most as many edges as P. This can always be guaranteed by simply swapping the reactant and product. Furthermore, define for any graph A, and node i in that graph and radius r the subgraph $A_{i}\left(r\right)$ as the subgraph of A containing all nodes that are at most r edges away from node i.

**FIGURE 2:**
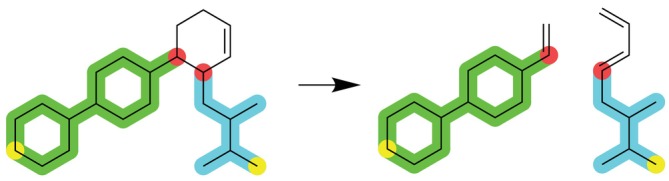
Illustration of the reaction mapping algorithm. The first step identifies the largest matching subgraph, shown in green and expanded from the yellow carbon. Next, a slightly smaller subgraph—one radius less, excluding the red atom—is used to map the atoms. Once the green subgraph is established, the algorithm finds the next largest unique matching subgraph, highlighted in cyan. Without matching the green subgraph first, the cyan subgraph could have been matched wrongly. The remaining unmapped atoms are part of the active center.

The algorithm begins by defining the radius r as half of the graph diameter. It then iterates for over every atom i in the reactant over every atom j in the product. If $R_{i}\left(r\right)$ and $P_{j}\left(r\right)$ are isomorphic, a mapping between $R_{i}\left(r-1\right)$ and $P_{j}\left(r-1\right)$ is computed and applied. The mapping is also applied to any terminal hydrogens connecting to any of the mapped atoms. Any atoms with a mapping applied to them are omitted in current or further iterations. After iterating over all atoms, r is reduced by one and the process repeats until r reaches two. This produces a mapping of all atoms that are not part of the active center of the reaction, as illustrated in Figure 2.

This leaves the active center of the reaction unmapped. If the upper bound of possible solutions is small enough, the algorithm iterates over all mappings with consistent elements and picks the best one. Otherwise, the algorithm continues with a bond‐breaking approach. It first iteratively tries to break bonds until every connected part of R can be mapped to a unique part of P, starting out with no broken bonds, and steadily increasing the amount of broken bonds. Once the correct breaking bonds are found, it iterates over all possible forming bonds until an isomorphism between R with broken and formed bonds, and P is found. The main computational step in this approach is the iteration through combinations of broken bonds. Per combination, a series of subgraph isomorphism checks need to be performed. In particular, concluding that no subgraph isomorphism exists can be a very expensive task. Chemically informed heuristics are used to limit the amount of combinations, as well as picking the most promising combinations first.

**FIGURE 3:**
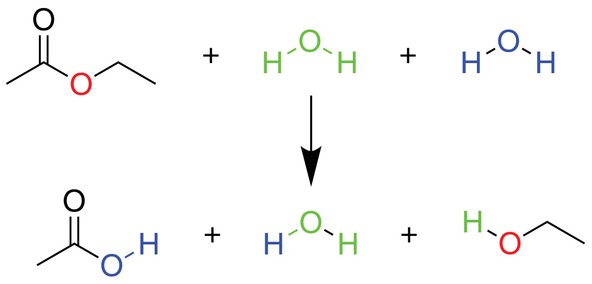
Water assisted hydrolysis of methyl acetate.

Additionally, the algorithm can be guided by giving input on some or all of the bonds that are known to be broken or formed, effectively providing part of the solution to the algorithm. This also opens up the possibility to force a non‐ideal solution, where more than the minimal amount of bonds breaks. This is useful for example in the case of solvent assisted hydrogen transfer, like water assisted hydrolysis as depicted in Figure 3.

In this case, the ideal solution for the atom‐mapping problem would leave one of the water molecules untouched, since the minimal way to get from the reactant to the product is to hydrolyze the ester bond with one water molecule. However, by forcing the breaking of two water molecules, the algorithm will find a solution where both water molecules partake.

### 2.2 Force Field Construction and Interpolation

**FIGURE 4:**
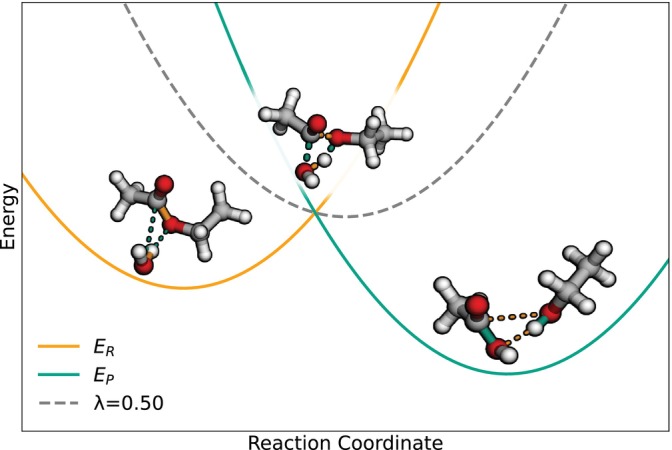
Illustration of force field interpolation between reactant and product. The shown structures correspond to optimized structures at the respective potentials. Green and orange (dashed) bonds indicate bonds that are forming and breaking respectively.

The first step in creating interpolated force fields is to generate separate force fields for the reactant and product. Given an input structure in SMILES string (or *XYZ*) format, the connectivity is determined based on covalent radii, after which all atom types of the second generation general Amber force field [46, 47] (GAFF 2) are automatically assigned as described in Ref. [48]. Any parameters for non‐bonded interactions that are not available in GAFF 2 are supplemented with universal force field (UFF) parameters [49]. Partial charges are by default calculated using D4 dispersion charges [50]. Optionally, these can be improved with RESP charges [51]. By default, force constants for unknown bonds and angles such as those involving metal centers are guessed with a default value of $2.5\times 10^{5}$ kJ/mol/nm^2^ and 1,000 kJ/mol/rad^2^ respectively. It is possible to reparameterise these force constants with the Seminario method [52] if desired, which will employ a subsystem Hessian calculation where only the required submatrices are calculated, speeding up the Hessian calculation significantly for larger systems. This then gives an energy expression for the reactant $E_{R}$ and product $E_{P}$ of the form

(1) $$\begin{array}{l} E_{i}\left(X\right)=\sum_{j\in i}^{\text{bonds}}U_{\text{bond}}^{\left(j\right)}\left(r_{j}\right)+\sum_{j\in i}^{\text{angles}}U_{\text{angle}}^{\left(j\right)}\left(\theta_{j}\right)+\sum_{j\in i}^{\text{torsions}}U_{\text{torsion}}^{\left(j\right)}\left(\phi_{j}\right)\\ +\sum_{j\in i}^{\text{impropers}}U_{\mathrm{imp}.}^{\left(j\right)}\left(\phi_{j}\right)+\sum_{j\in i}^{\text{pairs}}\left(U_{\text{Coul}.}^{\left(j\right)}\left(r_{j}\right)+U_{\mathrm{LJ}}^{\left(j\right)}\left(r_{j}\right)\right) \end{array}$$

where X are all coordinates of the system. The bond stretching term for breaking and forming bonds is modeled with a Morse potential, while other bond stretching and angle bending terms are defined by harmonic potentials. Furthermore, the proper and improper torsions are defined by Fourier series potentials, and the non‐bonded potential is a sum of Lennard‐Jones and Coulomb interactions. The interpolated potential V defined as

(2) $$V\left(X;\lambda \right)=\left(1-\lambda \right)E_{R}\left(X\right)+\lambda E_{P}\left(X\right)$$

then couples the reactant and product potential energy surface, where the parameter λ dials in the influence of the reactant and product as illustrated in Figure 4. For any constant value of λ between 0 and 1, V then becomes an approximation for the PES at a specific location along the reaction pathway. This potential is inspired from the mapping potential in the EVB method [28]. To prevent this value from being dominated by non‐bonded interactions for systems that closely resemble either the reactant or product, a soft‐core potential is used to model the non‐bonded interactions of the breaking and forming bonds. The details of this soft‐core potential are given in the Supporting Information.

### 2.3 Sampling

This definition for V produces reasonable relative energies along the entire reaction, but does not necessarily produce accurate structures at intermediate values of λ. To improve these, the structure mapping potential $\tilde{V}$ is constructed. In this potential, the terms involving bonds that are breaking or forming are modeled across the entire reaction as harmonic potentials, which replaces the combination of Morse and soft‐core non‐bonded interactions. In the broken state, these harmonic potentials have an equilibrium distance set to the Lennard‐Jones equilibrium distance, and a scaled force constant of 0.2k. The 1–3 and 1–4 interactions for the remaining non‐bonded interactions are then calculated based on the union of the bonds present in the reactant and product. Furthermore, to account for donor‐acceptor distance compression in the transition state, the equilibrium distance of forming and breaking bonds is scaled down to $\tilde{r}$ for intermediate values of λ as

(3) $$\tilde{r}\left(\lambda ;p\right)=\Delta r{\left(\frac{r\left(\lambda \right)-r_{\min.}}{\Delta r}\right)}^{p}+r_{\min.}$$

(4) $$r\left(\lambda \right)=\left(1-\lambda \right)r_{R}+\lambda r_{P}$$

where $r_{R}$ and $r_{P}$ are the equilibrium distances for the reactant and product, Δr and $r_{\min.}$ are the difference and minimum of these values, and p is a parameter which can be tuned to control the tightness. A value of p=3.25 has been used for this work as it has been found to produce reliable results. Similarly, intermediate values of force constants for forming and breaking bonds $\tilde{k}$ are also scaled down as

(5) $$\tilde{k}\left(\lambda ;\gamma \right)=k\left(\lambda \right)\left(\gamma +\left(1-\gamma \right){\left(2\lambda -1\right)}^{2}\right)$$

(6) $$k\left(\lambda \right)=\left(1-\lambda \right)k_{R}+\lambda k_{P}$$

where $k_{R}$ and $k_{P}$ are the force constants for the reactant and product, and γ is a parameter to control the scaling of the force constant, which is taken as γ=0.8 for this work. Future work could investigate an empirical or theoretically supported derivation for the value of p and γ.

The system is then sampled over a range of discrete values of λ between 0 and 1. By default, 21 evenly spaced values of λ are used. Starting out with the reactant input structure (λ=0), the molecular structure is optimized and then sampled for a short time with molecular dynamics at 600 K in an NVT ensemble, after which the structure is optimized again. This is done with the structure mapping potential $\tilde{V}\left(X;\lambda \right)$. The final structure is saved, and the MM energy is then calculated with $V\left(X;\lambda \right)$. The structure is then used as input for the next value of λ. This procedure is repeated for every value of λ up to λ=1. It is possible to reverse the direction of the scan, or to perform the scan in both directions. It is also possible to perform a high temperature conformational sampling at some or all values of λ instead of just a single optimisation. Any conformational structures that belong to the same value of λ with an energy of less than 0.1 kJ/mol are discarded. This produces a range of molecular structures and corresponding energies with one or more structures per value of λ. Optionally, the QM energy of these structures can be calculated automatically on any desired level of theory for a specified amount of lowest lying conformers. Per value of λ, the conformer with the lowest MM energy is collected (or QM energy if this is calculated). Of these collected structures, the one with the highest energy is selected as a guess for the transition state structure.

The entire workflow as well as the visualization of the results is implemented in VeloxChem [35], using NetworkX [53] for graph handling and OpenMM [54] for the molecular dynamics. It can be ran with just a few lines of Python code as demonstrated below.import veloxchem as vlxethyl_acetate = vlx.Molecule.read_smiles('CC(=O)OCC')water = vlx.Molecule.read_smiles('O')acetic_acid = vlx.Molecule.read_smiles('CC(=O)O')ethanol = vlx.Molecule.read_smiles('OCC')tsguesser = vlx.TransitionStateGuesser()*# Optional settings*tsguesser.scf_scan = Trueresults = tsguesser.find_transition_state(    [ethyl_acetate, water],    [acetic_acid, ethanol],)tsguesser.show_results(results)


**FIGURE 5:**
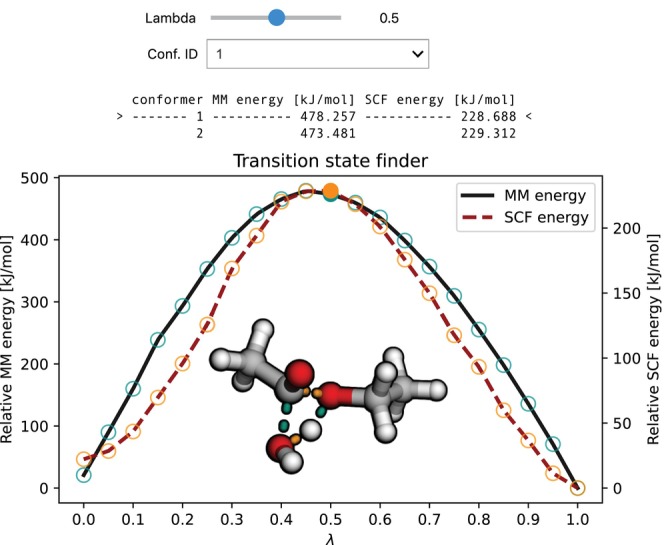
The interactive transition state guess selection widget in VeloxChem.

The results object is a dictionary that is automatically saved in HDF5 format to disk and can be loaded for later use. The last line produces an interactive Jupyter widget as shown in Figure 5, where the user can inspect the various generated transition state guesses, their energies and visualize the structures in 3D and select the best candidate if the default choice is not satisfactory. The selected structure can then be easily extracted in *XYZ* format for optimisation with any software.

## 3 Results and Discussion

### 3.1 Examplary Systems

To illustrate its applicability, the workflow was applied to the following reactions:

1. Hydrogen transfer from Ref. [55]
2. Proton transfer from Ref. [56]
3. Proton shuttle from Ref. [57], making use of the forced breaking and forming bonds feature
4. Alder‐ene reaction from Ref. [58]
5. An inter‐molecular S_N_2 reaction from Ref. [59]
6. Double esterification followed by the double hydrolysis of morphine, giving 4 transition states
7. The transition states for both regiomers of a 4 + 2 cycloaddition from the total synthesis of Taiwaniadduct J from Ref. [60]. Correct regiomeric TS guesses were selected manually from the best λ value
8. Macro‐molecular tricyclic ring‐opening part of an S_N_1 mechanism from Ref. [61]
9. An azide‐amine cycloaddition in a molecular capsule from Ref. [62]
10. Pd‐mediated Mizoroki–Heck catalytic cycle for coupling chlorobenzene with ethene, giving 5 transition states

**FIGURE 6:**
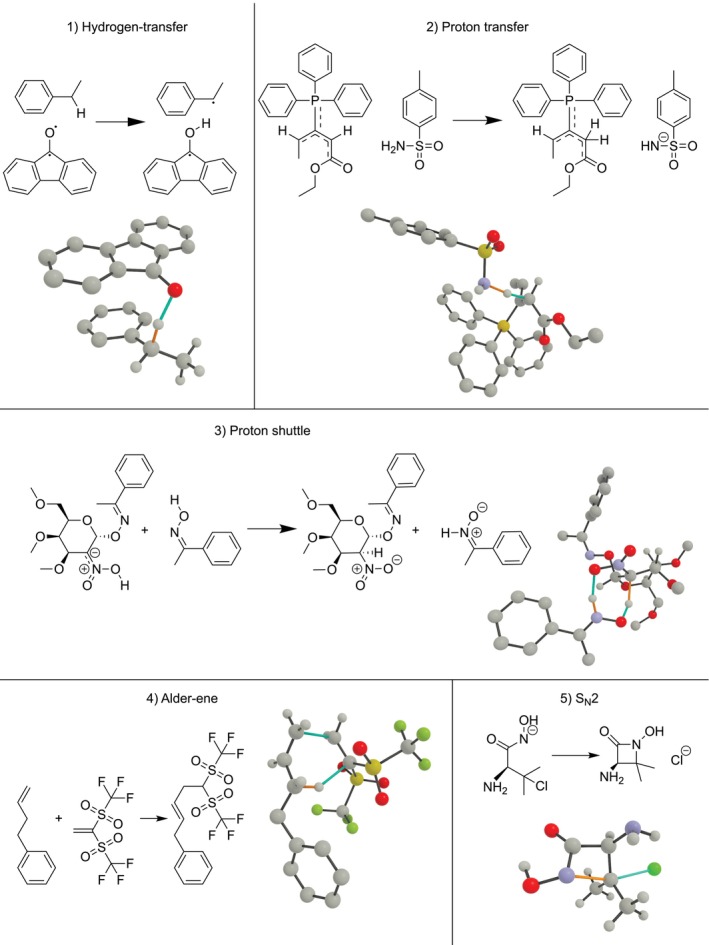
Reactions and TS structures of system 1–5. Forming bonds are colored green and breaking bonds are colored orange. Hydrogens on distant groups have been omitted for clarity. All visualizations were done with VIAMD [63].

**FIGURE 7:**
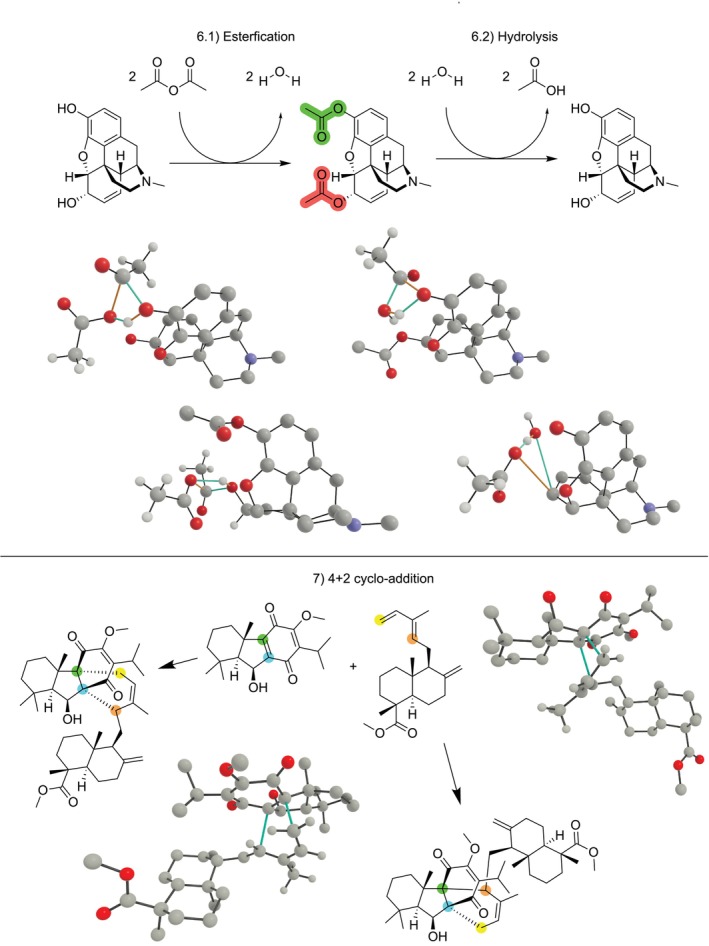
Reactions and TS structures of system 6 and 7. System 6 consists of four reactions in which esterification of the green group occurs first, followed by esterification of the red group, after which hydrolysis of the green group occurs, followed by hydrolysis of the red group.

**FIGURE 8:**
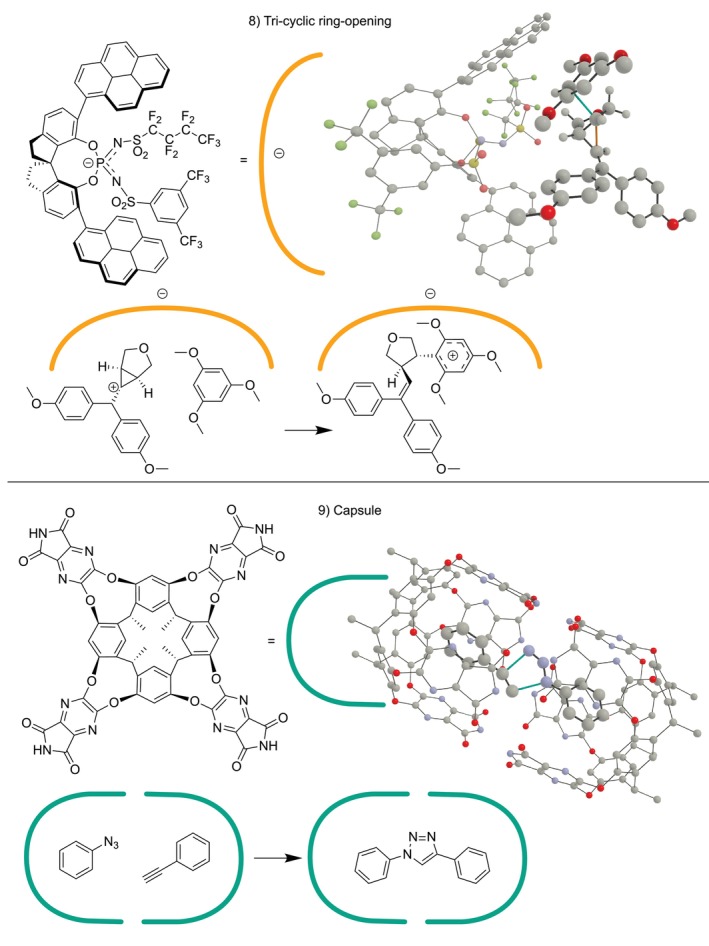
Reactions and TS structures of system 8 and 9.

**FIGURE 9:**
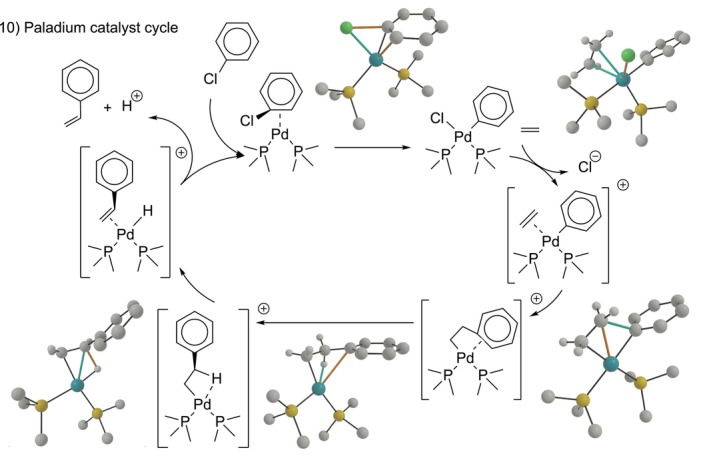
Reactions and TS structures of system 10.

All reactions and their optimized structures are shown in Figures 6, 7, 8, 9. All TS guesses were obtained from SMILES codes, except for reaction 8, 9 and 10 where optimized structures were used as input. For all guesses, conformer searches were performed both in the forward and backward direction. All optimizations were performed at the PBE0‐D4/def2‐SVP level of theory, and the palladium in system 10 was modeled with def2 effective core potentials. For most reactions, the obtained guess immediatly converged to a transition state, after which frequency calculations verified a single imaginary frequency. For reaction 3, tight convergence criteria were necessary for convergence, and for reaction 9, a second imaginary frequency was found after the first optimization, which was eliminated by increasing the grid level to 5. For the rearrangement in reaction 10, the automatic guess at λ=0.45 did not converge, but a manual selection of the guess at λ=0.55 did converge. Optimizations converged with an average of 108 gradient calculations, with the substitution step in the Pd‐catalytic cycle being the easiest to converge with just 13 steps and the β‐hydride elimination in the Pd‐catalytic cycle being the most difficult with 280 optimization steps. All optimizations were started with an analytical Hessian and performed with default VeloxChem settings. For detailed computational details on the optimisation settings, the reader is referred to the Supporting Information.

### 3.2 Benchmark Results

The workflow was also applied to a set of 121 reactions obtained from Ref. [18] to benchmark its accuracy and performance, and compare this to the accuracy and performance of the NEB‐TS method. The set is in turn constructed from the combination of reaction sets from Ref. [10, 17]. The set encompasses a wide variety of reaction classes, ranging from common substitutions, transfers, eliminations, and cycloadditions to more complex rearrangements involving up to six simultaneously forming or breaking bonds. The majority of reactions are organic; of the inorganic reactions, 13 feature aminoboranes, five involve silicon, and one includes magnesium. Two reactions involving a double bond rotation have been excluded, and three reactions that were included in either Ref. [17] or Ref. [10] but not in Ref. [18] have also been included. In the five reactions that have non‐participating ligands, these ligands were removed. Although Ref. [17] claims that all reactions in their set are elementary steps, Ref. [18] shows at least one example where this is clearly not the case, and we expect that this is the case for some other reactions as well. The reader is referred to the Supporting Information for a comprehensive overview of the dataset as well as all structures. All TS guesses were started from the *xyz* structures supplied in the set. Backward and forward scanning was performed with conformational sampling, after which the QM energy was calculated with density functional theory (DFT) at the B3LYP‐D4/def2‐SVP level of theory, from which the best structure was automatically selected. This guess was optimized with B3LYP‐D4/def2‐SVP (the same as in Ref. [18]), after which an intrinsic reaction coordinate (IRC) analysis was performed. The reference structures were also re‐optimized with the same level of theory, and an IRC analysis was performed on these as well. All computations were performed with the VeloxChem program [35]. This approach allows for direct comparison with the NEB‐TS method.

In the following section, any mention of reference will refer to Ref. [18]. Furthermore, TS guess refers to the guessed structure produced with the workflow from this work, optimized TS refers to the TS structure obtained after optimization of the TS guess, and reference TS refers to the TS structure from the reference. For discussing the IRC results, the IRC analysis is considered to have succeeded if the correct reactant and product are found at the connecting minima.

For 91 reactions, the workflow from this work produced a TS guess for which the optimization converged to a TS with one imaginary frequency and correct IRC. For two reactions, the TS guess did not converge to a TS after 300 optimization steps, and in four more cases, either none or more than one imaginary frequency was found.

In all cases for the reference, the re‐optimization of the TS led to a saddle point that did not significantly differ from the original structure. These TSs produced one imaginary frequency and a correct IRC in 96 reactions. One reference TS produced two imaginary frequencies.

In the case of 24 optimized TSs, the IRC analysis failed. This was also the case for 24 reference TSs. This is in contradiction with the claim in the reference that all structures produce correct IRCs. This is likely at least in part due to differences in the optimization algorithm used for the IRC calculation, which will be discussed later. However, for only 15 of these reactions, both the optimized TS and the reference TS demonstrated a failing IRC analysis. On average over all reactions, 47 SCF energy calculations were performed during the scan, and 60 gradient calculations were required to perform the geometry optimisation of the guess. This stands in contrast to the average of 305 gradient calculations per reaction required by NEB‐TS. For a comparable accuracy level, the currently presented method requires a significantly lower computational cost on this dataset. A summary of the results can be found in Table 1, whilst a detailed overview of the results is contained in Table S1 of the Supporting Information of this article as well as *xyz* files for all structures.

**TABLE 1:** Summary of the results from the benchmark set.

	This work	Ref. [18]
Success	91	96
Opt. failure	2	0
Wrong freq.	4	1
IRC failure	24	24

**FIGURE 10:**
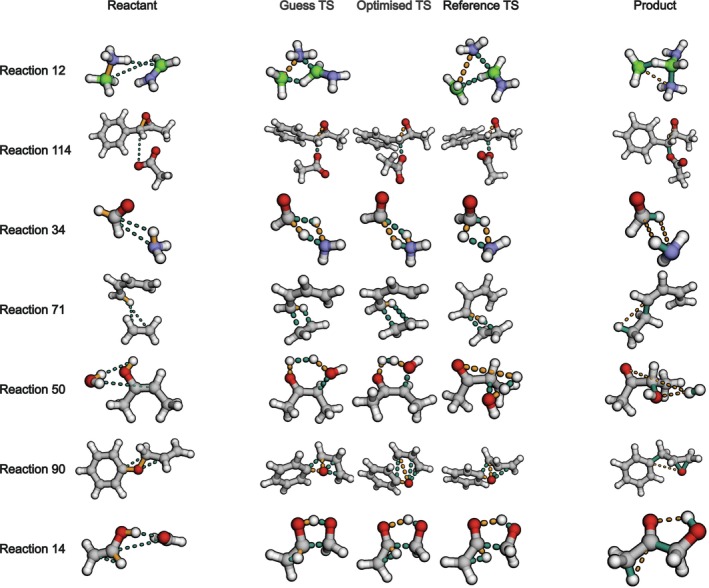
An overview of some reactions representing the main success and failure cases from the benchmark set. Green and orange (dashed) bonds are forming and breaking bonds respectively. The guess for reaction 12 did not converge to a transition state. In reaction 114, $\Delta E^{‡}=8$ kJ/mol, while for reaction 34, $\Delta E^{‡}=-34$ kJ/mol. For reaction 71, the optimized TS fails the IRC, despite the guess being initially similar to the reference structure. Reaction 50 produces the correct IRC for the optimized guess, but not for the reference structure. Reaction 90 is an example of a difficult rearrangement for which both this work and the reference fail the IRC correctly. Reaction 14 shows an example where the IRC fails for both this work and the reference, despite having found the same structure.

This section will first discuss the energetics and structure of all successfully converged cases, followed by a discussion on the cases in which the optimizations or frequency calculations failed. The section is concluded with an analysis on the failure cases of the IRCs, and comparing those to the reference. Some selected reactions representing the main success and failure cases from the benchmark set are shown in Figure 10. The numbers of the reactions mentioned in this section correspond to those in the Supporting Information.

For discussing energetics, the energy difference $\Delta E^{‡}$ is defined as

(7) $$\Delta E^{‡}=E_{\mathrm{opt}.}^{‡}-E_{\mathrm{ref}.}^{‡}$$

with $E_{\mathrm{opt}.}^{‡}$ being the energy of the optimized TS structure, and $E_{\mathrm{ref}.}^{‡}$ being the energy of the reference TS. In the far majority of cases where both the reference and this work had converging IRCs, $|\Delta E^{‡}|\;<1$ kJ/mol. In some of these cases the molecular conformation was slightly different with ligands more distant from the active center pointing in different directions.

For all eight cases for which $\Delta E^{‡}<-1$ kJ/mol, the active center was cyclic. In all cases, differences in conformations lead to differences in energy, where larger distortions of angles and torsions lead larger energy differences. Two outliers both involved formaldehyde, namely the H_2_ elimination of formaldehyde (reaction 113) with $E_{\mathrm{opt}.}^{‡}=67.8$ and $E_{\mathrm{ref}.}^{‡}=76.0$ kJ/mol, and the C—H/N—H transfer between formaldehyde and ammonia (reaction 34) with $E_{\mathrm{opt}.}^{‡}=71.6$ and $E_{\mathrm{ref}.}^{‡}=111.2$ kJ/mol. On the other hand, the four cases for which $\Delta E^{‡}>1$ kJ/mol all had non‐cyclic active centers. Here, longer ligands with significant conformational freedom led to structural and energetic differences, such as for the addition of SiOCl_2_ to 2‐butene‐1‐ol (reactions 86 and 87). The largest outlier, an oxirane ring‐opening (reaction 114) had $E_{\mathrm{opt}.}^{‡}=28.4$ kJ/mol whereas $E_{\mathrm{ref}.}^{‡}=21.24$ kJ/mol. Although the error for B3LYP with a double zeta basis set is of the order of 10 kJ/mol for reaction barriers [64], this error is below 5 kJ/mol when comparing relaxed optimized conformers of the same molecule [65].

**FIGURE 11:**
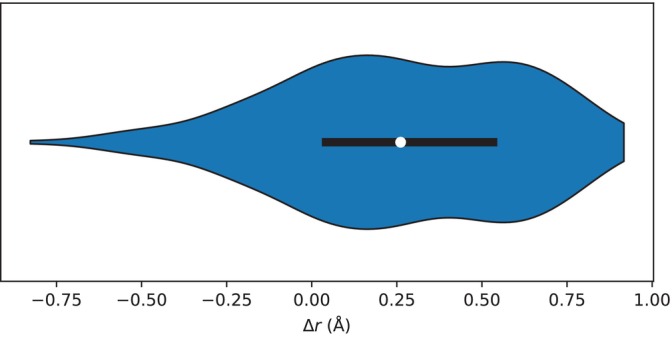
Violin plot of the difference between the bond length for the TS guess and optimized TS for all breaking or forming bonds for all successfully optimized TSs with correct IRCs (94 structures, 277 total bonds). The white dot denotes the median at 0.26 Å, and the black bars denote the first and third quartile. The average is 0.26 Å.

For the TSs with correct IRCs, the geometrical difference between the TS guess and the optimized TS was analyzed. For each breaking or forming bond, the difference in bond length between the TS guess and the optimized TS was calculated. The results are shown in Figure 11. For the TS guesses, the breaking and forming bonds are on average 0.26 Å longer in the guessed structure than in the optimized TS, despite the fact that the bond lengths had already been scaled down as per Equation (3).

Even though part of the reactions from the set were only partly or not at all described by GAFF, the workflow in this paper still produced good TS structures in some of these cases. In particular, the aminoborane reactions (reactions 1–13 and 119–121) all are not at all described by GAFF, and yet still converged to saddle points in 12 out of 13 cases. Their IRCs failed for six of these cases. However, this was also the case for seven reference TS structures partly corresponding to different reactions, highlighting the general difficulty of these reactions.

The two cases in which the optimisation of the TS guess failed are the H_2_ elimination from ethane (reaction 30) and a complex aminoborane reaction (reaction 12) where the product contains a bridging hydrogen bonding to two boron atoms. In both these cases, the reference TS structures had correctly converging IRCs. In a similar fashion, the optimized TS produced no imaginary frequencies for the Cl_2_ elimination from SO_2_Cl_2_ (reaction 65). And for an internal OH transfer reaction (reaction 93), the optimized TS produced three large imaginary frequencies. These cases highlight some of the limitations of a force‐field based approach. Furthermore, one taxadiene carbocation proton transfer (reaction 97) produced a second small imaginary frequency, whereas the reference had a failing IRC. The optimized TS and reference TS were also significantly different in this case. Two other carbocation proton‐transfers had converging optimized TSs with correct IRCs, whereas the reference TSs had failing IRCs. Lastly, for the Grignard addition to benzophenone (reaction 109), both this work and the reference found the same structure that contained a small second frequency.

When discussing IRC failures, four cases can be distinguished: where only the optimized TS fails (nine reactions), where only the reference TS fails (eight reactions), where both fail with the same structure (six reactions), and where they fail with qualitatively different structures (nine reactions). For the reactions where the optimized TS did not converge to a correct IRC, there were always three or more bonds changing. In some cases, the optimized TS guess is reasonably close to the reference TS but ends up optimizing to a significantly different lower lying saddle point, such as with a butadiene ethylene rearrangement (reaction 71). This was also the case for two reactions involving an H_2_ elimination (reactions 64 and 110) where the hydrogen molecule was ejected during the TS optimisation. It can be reasonably assumed that in the majority of these cases, the guess was simply not suitable. In some other rearrangement reactions, the guess was close to the final TS but converged to another closely located saddle point belonging to a different reaction path, such as with another butadiene ethylene rearrangement (reaction 78). For the reactions where the reference failed to the IRC while this work succeeded, there were between two and four bonds changing. These cases have not been investigated in further detail.

In cases where both IRCs fail with different structures, the reactions are all difficult rearrangements with either four or five bonds changing. For the epoxide formation of allyl phenyl ether (reaction 90), Ref. [18] showed that it is not a multi‐step reaction. However, the IRC found in this work on this reference structure does not converge to the same intermediate as reported. It might be the case that more of these reactions are multi‐step reactions, but this has not been investigated. In all cases, $\Delta E^{‡}<0$, highlighting how the workflow in this work has a tendency to fall into (possibly incorrect) lower lying pathways.

Lastly, the six cases where this work and the reference produced the same TS and yet failed to converge the IRC are also all difficult rearrangements with three to five changing bonds. In all cases, the structures look reasonable. In the case of a tricyclic ring‐formation from cis‐butadiene and ethylene (reaction 76), a local minimum might have been found in the IRC. In the two other cases (reaction 14 and 31), an incorrect part of the system is fully ejected during one side of the IRC. For three butadiene‐ethylene rearrangements (reactions 77, 79 and 81), the IRCs converged to trans‐butadiene instead of cis‐butadiene. Lastly, the ethyl‐transfer from ethyl‐acetate to ammonia (reaction 26) produces an optimized TS that is identical to the reference TS, and the IRC produces the correct product, but with the wrong atom ordering in both cases.

In comparing the convergence of IRCs, it should be noted that this work through its implementation in VeloxChem relies on the optimizer of geomeTRIC [66], which uses a quasi‐Newton method with a Hessian update for the IRC optimisation. The results reported in the reference were produced with ORCA [18] which uses steepest descent for its IRC [67] with an optional correction term. While the ORCA manual does not specify the exact details of the coordinate scheme used, it is likely that this is different from geomeTRIC as well. As shown in Ref. [66], the chosen coordinate scheme for performing the optimization has significant impact on the convergence behavior. Both these details are expected to have a significant impact on the convergence of the IRC in the case of more complex PES landscapes. This is especially the case when there are other saddle points and/or intermediate minima in close proximity of the correct saddle point that belong to a different IRC. This is for example the case with some of the aminoborane reactions (reactions 1–13 and 119–121) and cis‐butadiene + ethylene reactions (reactions 69–81).

## 4 Conclusion

A force field interpolation method for generating transition state guesses has been presented. The complete workflow is demonstrated on ten diverse chemical systems. These examples illustrate the method's robustness and versatility, encompassing a range of reaction classes such as proton‐transfers, an $S_{N}2$ reaction, and various hydrolysis and esterfication reactions. The set also includes larger systems up to 250 atoms, and examples featuring heavier atoms including a full Pd‐mediated catalytic cycle. In all cases, transition state guesses were obtained in a low number of optimization steps. Furthermore, the interactive Jupyter notebook widget enables intuitive refinement of initial guesses, allowing chemists to actively improve them using domain‐specific chemical insight.

The method is also benchmarked on a set of 121 small systems. In 115 cases, a transition state with one imaginary frequency was found, with an average of 107 gradient calculations. In 24 of these cases, IRC analyses did not produce the correct reactant and product. In some cases, this is caused by the wrong transition state being found. However, in some other cases, structures that were found were similar or identical to those reported in other studies where they were claimed to have converging IRCs [18]. In yet some other cases, the guesses from this workflow converged to transition states that produced correct IRCs, whereas the reference structures did not produce the correct IRCs. This indicates that for more difficult reactions, differences in the implementation details of the IRC procedure can lead to qualitatively different results.

These results are surprising, as there is no direct physical reason why simple interpolation of force fields yields structures that are accurate enough to be successfully optimized to a TS. Furthermore, for this method to function predictably, a force field needs to describe the system well enough. Even though systems like aminoborane reactions (reactions 1–13 and 119–121) showed good results despite their lacking description in GAFF 2, care must be taken in transferring this method to other systems that are not accurately described in GAFF 2. This was also illustrated by the Paladium example which required one reaction to be manually tweaked before convergence. However, this shortcoming is transparent in the sense that it is known a priori if a specific system is or is not described by a force field, in contrast to some machine‐learning based approaches. Future developments on the program will include the ability to reparameterise force fields with QM‐hessian data to alleviate this shortcoming as well as support for other force fields.

Overall, the presented workflow performed similarly to NEB‐TS on the benchmark set, and in a couple select cases, lower lying transition states were found. This all while requiring a significantly lower computational cost. The presented work is integrated in the open source VeloxChem program. An accompanying demonstrative Jupyter notebook is provided in the Supporting Information, providing an accessible starting point for any interested user. For more information, the reader is referred to the eChem book [68].

## Funding

This work was supported by the Swedish Research Council (2023‐5171).

## Supporting information


**Table S1:** Overview of errors, amount of changing bonds, barrier energies and imaginary frequencies for all reactions for all optimized TSs and reference TSs.

## Data Availability

The data that support the findings of this study are openly available in SI xyz‐structures at https://doi.org/10.5281/zenodo.20424591.
