# Supplementary material for: From SMILES Codes for Reactants and Products to Transition States With VeloxChem
Source: J Comput Chem. 2026 Jul 3;47(18):e70454. doi: 10.1002/jcc.70454 (PMC13332112; doi:10.1002/jcc.70454)
Supplement: Supplementary file 1 — Table S1: Overview of errors, amount of changing bonds, barrier energies and imaginary frequencies for all reactions for all optimized TSs and reference TSs. [file JCC-47-0-s001.pdf]

From SMILES Codes for Reactants and Products to Transition States with VeloxChem

Bastiaan van Hoorn,* Patrick Norman,* and Mårten S. G. Ahlquist

Division of Theoretical Chemistry and Biology, School of Engineering Sciences in Chemistry, Biotechnology and Health, KTH Royal Institute of Technology, SE-100 44 Stockholm, Sweden

E-mail: bvh@kth.se; panor@kth.se

Contents

Soft core interaction formulation

The following soft core formulation for the Lennard-Jones and Coulomb interactions is used to prevent diverging values for the mapping potential V. It is applied only to the non-bonded interactions of the breaking and forming bonds.

$$U_{\mathrm{LJ}}(r) = \begin{cases} 4\varepsilon_{jk}\left(\frac{\sigma_{jk}^{12}}{r^{12}} - \frac{\sigma_{jk}^{6}}{r^{6}}\right) & , r \geq r_{\mathrm{LJ}} \\ 4\varepsilon_{jk} U_{\mathrm{LJ,lin}}(r) & , \ r < r_{\mathrm{LJ}} \end{cases} \tag{1}$$

$$U_{\mathrm{LJ,lin}} = \left(\frac{78\sigma_{jk}^{12}}{r_{\mathrm{LJ}}^{12}} - \frac{21\sigma_{jk}^{6}}{r_{\mathrm{LJ}}^{6}}\right)\frac{r^2}{r_{\mathrm{LJ}}^2} - \left(\frac{168\sigma_{jk}^{12}}{r_{\mathrm{LJ}}^{12}} - \frac{48\sigma_{jk}^{6}}{r_{\mathrm{LJ}}^{6}}\right)\frac{r}{r_{\mathrm{LJ}}} + \left(\frac{91\sigma_{jk}^{12}}{r_{\mathrm{LJ}}^{12}} - \frac{28\sigma_{jk}^{6}}{r_{\mathrm{LJ}}^{6}}\right) \tag{2}$$

$$U_{\mathrm{Q}}(r) = \begin{cases} \frac{q_j q_k}{4\pi\varepsilon_0 r}, & r \geq r_{\mathrm{Q}} \\ \frac{q_j q_k}{4\pi\varepsilon_0}\left(\frac{r^2}{r_{\mathrm{Q}}^3} - \frac{3r}{r_Q^2} + \frac{3}{r_Q}\right), & r < r_{\mathrm{Q}} \end{cases} \tag{3}$$

$$r_{\mathrm{LJ}} = \alpha_{\mathrm{LJ}}\left(\frac{26}{7}\sigma_{jk}\right)^{1/6} \tag{4}$$

$$r_{\mathrm{Q}} = \alpha_{\mathrm{Q}}(1 + |q_j q_k|) \tag{5}$$

This formulation replaces the original potential with a harmonic potential for $r < r_{\mathrm{LJ}}$ and $r < R_{\mathrm{Q}}$ for the Lennard-Jones and Coulomb potential respectively. This produces a finite energy for $r = 0$. The parameters α_{LJ} and α_{Q} scales till which radius the soft-core potential is active. For this report is set to $\alpha_{\mathrm{LJ}} = 0.85$, $\alpha_{\mathrm{Q}} = 0.3$.

Optimisation details

All optimisation calculations were performed with standard VeloxChem settings:

1. DFT grid level: 4

2. SCF energy convergence threshold: 1e-6 Hartree
3. Optimisation energy change threshold: 1e-6 Hartree
4. Gradient RMS threshold: 3e-4 Hartree/Bohr
5. Gradient Max threshold: 4.5e-4 Hartree/Bohr
6. Displacement RMS threshold: 1.2e-3
7. Displacement Max threshold: 1.8e-3
8. Initial trust radius: 0.01 Bohr
9. Maximum trust radius: 0.03 Bohr

Dataset details

In this section a summary of the results on the full benchmark as well as all details in its construction are presented, shown in Table S1. The Fail and Ref fail columns indicate whether the optimisation or IRC calculation failed for tho optimised TS or the reference TS respectively, where "OPT" indicates optimisation failure, "FRQ" indicates not exactly one imaginary frequency was found, and "IRC" indicates IRC failure. The Note column indicates some information as follows:

(a) Originally from Ref. 17

(b) Originally from Ref. 10

(c) Not included in dataset from Ref. 18

(d) Removed molecule that was passively present

(e) Identical structure despite IRC failure

(f) Forms trans-butadiene instead of cis-butadiene

(g) IRC produces correct molecular structure but with the wrong atom ordering

The bonds column indicates the amount of bonds changing and forming in the reaction. Reaction 88 and 89 have been ommitted because they are excluded from the dataset. The numbering is kept as close as possible to the numbering from Ref. 18.

Table S1: Overview of errors, amount of changing bonds, barrier energies and imaginary frequencies for all reactions for all optimised TSs and reference TSs.

ID.	Reaction	Fail	Ref fail	Note	Bonds	$E^{\ddagger}_{\text{opt.}}$ kJ/mol	$E^{\ddagger}_{\text{ref.}}$ kJ/mol	$\Delta E^{\ddagger}$ kJ/mol	ω_0 cm^{-1}	$\omega_{0,\text{ref.}}$ cm^{-1}
1	$2\,NH_2BH_2 \rightarrow NH_2BHNH_2BH_3$			a	3	9.32	9.33	-0.01	345	345
2	$2\,NH_2BH_2 \rightarrow NH_2BHNH_2BH_3{}^{i}$	IRC		a	5	9.33	88.21	N/A	344	928
3	$2\,NH_2BH_2 \rightarrow NH_3BH_2NHBH_2$	IRC		a	3	29.29	28.52	N/A	749	1247
4	$2\,NH_2BH_2 \rightarrow 2\,NH_2BH_2{}^{i}$			a	4	93.71	91.48	2.23	2482	2459
5	$2\,NH_2BH_2 \rightarrow 2\,NH_2BH_2{}^{ii}$			a	4	67.64	67.64	0.00	2580	2581
6	$2\,NH_2BH_2 \rightarrow 2\,NH_2BH_2{}^{iii}$	IRC	IRC	a	4	17.69	19.87	N/A	201	434
7	$2\,NH_2BH_2 \rightarrow NH_3BH_2BHNH_2$			a	3	53.88	53.88	0.01	1492	1489
8	$NH_2BH_2 + NH_3BH_3 \rightarrow NH_3BH_2NH_2BH_3$			a	3	35.69	35.69	0.00	216	216
9	$NH_2BH_2 + NH_3BH_3 \rightarrow NH_3{+}NH_2B_2H_5$		IRC	a	4	26.77	32.19	-5.42	437	164
10	$NH_3BH_3 \rightarrow NH_2BH_2 + H_2$			a, d	3	38.28	38.28	0.00	1381	1382
11	$NH_2BH_2 + NH_3BH_3 \rightarrow 2\,NH_2BH_2 + H_2$			a	5	37.01	37.01	0.00	1368	1369
12	$NH_3BH_3 + NH_2BH_2 \rightarrow NH_3BH_2NH_2$	OPT		a	3		36.47	N/A		213
13	$NH_3BH_3 + NH_2BH_2 \rightarrow NH_3 + NH_2BHBH_4$		IRC	a	4	28.72	32.19	-3.47	405	164
14	$CH_2CHOH + CH_2O \rightarrow OHCH_2C(CH_3)O$	IRC	IRC	a, e	5	56.31	56.31	N/A	1220	1212
15	$CH_2CHOH + CH_2O \rightarrow OCHCH(CH_3)OH$			a	3	50.55	50.55	-0.01	1217	1216
16	$CH_2CHOH + CH_2O \rightarrow OHCH_2CH_2CHO$			a	3	12.28	12.28	0.00	799	800

Continued on next page

Table S1: Overview of errors, amount of changing bonds, barrier energies and imaginary frequencies for all reactions for all optimised TSs and reference TSs. (Continued)

ID.	Reaction	Fail	Ref fail	Note	Bonds	$E^{\ddagger}_{\text{opt.}}$ kJ/mol	$E^{\ddagger}_{\text{ref.}}$ kJ/mol	$\Delta E^{\ddagger}$ kJ/mol	ω_0 cm^{-1}	$\omega_{0,\text{ref.}}$ cm^{-1}
17	$CH_2CHOH + CH_2O \rightarrow OCHCH_2CH_2OH$	IRC	IRC	a	5	86.93	98.11	N/A	2004	592
18	$CH_2CHOH + CH_2O \rightarrow CH_2CH_2 + OCHOH$			a	4	79.30	79.30	0.00	1127	1127
19	$CH_2CHOH + CH_2O \rightarrow OHCH_2C(CH_2)OH$		IRC	a	3	77.48	78.78	-1.30	2260	2131
20	$CH_2CHOH + CH_2O \rightarrow CH_2CHOCH_2OH$			a	3	66.69	66.69	0.00	722	722
21	$CH_2CHOH + CH_2O \rightarrow CH_2CHOCH_2OH$			a	3	38.50	38.53	-0.03	1747	1719
22	$CH_2CHOH + CH_2O \rightarrow CH_2CO + CH_3OH$			a	4	30.92	30.92	0.00	1290	1289
23	$CH_2CHOH + CH_2O \rightarrow CH_2CHOH + CH_2O$			a	4	82.94	82.92	0.02	2832	2860
24	$CH_2CHOH + CH_2O \rightarrow OHCH_2CH(CH_2O)O$			a	4	62.59	62.59	0.00	787	786
25	$CH_2CHOH + CH_2O \rightarrow O(C)CH_2CH(CH_2)OH$			a	2	71.60	72.01	-0.41	1254	1266
26	$CH_3CH_2OC(O)CF_3 + NH_3 \rightarrow OC(OH)CF_3 + CH_3CH_2NH_2$	IRC	IRC	a, g	4	56.01	56.00	N/A	536	536
27	$CH_3CH_2OC(O)CF_3 + NH_3 \rightarrow CH_3CH_2OC(O)CF_3^{i} + NH_3$			a, d	2	48.27	48.88	-0.61	499	508
28	$CH_3CH_2OC(O)CF_3 + NH_3 \rightarrow CH_3CH_2OC(OH)(NH_2)CF_3$			a	3	37.43	36.81	0.62	1618	1576
29	$CH_3CHO \rightarrow CH_2CHOH$			a	2	68.32	68.32	0.00	2165	2165
30	$CH_3CH_3 \rightarrow CH_2CH_2 + H_2$	OPT		a	3		120.31	N/A		2038
31	$CF_3CF(C)OCF_2 \rightarrow OCFCF(CF_3)F$	IRC	IRC	a, e	3	54.91	54.90	N/A	245	244
32	$CF_3CF(C)OCF_2 \rightarrow OCFC(CF_3)F_2$			a	3	54.30	54.30	0.01	402	401

Continued on next page

Table S1: Overview of errors, amount of changing bonds, barrier energies and imaginary frequencies for all reactions for all optimised TSs and reference TSs. (Continued)

ID.	Reaction	Fail	Ref fail	Note	Bonds	$E^{\ddagger}_{\mathrm{opt.}}$ kJ/mol	$E^{\ddagger}_{\mathrm{ref.}}$ kJ/mol	$\Delta E^{\ddagger}$ kJ/mol	ω_0 cm^{-1}	$\omega_{0,\mathrm{ref.}}$ cm^{-1}
33	$CF_3CF(C)OCF_2 \rightarrow FCF_2C(O)CF_3$			a	3	47.18	47.18	0.00	286	289
34	$CH_2O + NH_3 \rightarrow CH_2O + NH_3$		IRC	a	4	71.57	111.23	-39.66	988	1306
35	$CH_2O + NH_3 \rightarrow CH_3ONH_2$			a	3	99.49	99.49	0.00	1753	1758
36	$CH_2O + NH_3 \rightarrow NH_2CH_2OH$			a	3	32.30	32.30	0.00	1512	1512
37	$CH_3CHC(CH_3)OH \rightarrow CH_3CH_2C(CH_3)O$			a	2	53.87	53.87	0.00	2142	2141
38	$CH_3CHC(CH_3)OH \rightarrow CH_3CH_2C(CH_2)OH$		IRC	a	2	80.44	83.86	-3.42	1804	1564
39	$CH_3CHC(CH_3)OH \rightarrow CH_2CHCH(CH_3)OH$		IRC	a	2	83.61	82.67	0.94	1749	607
40	$CH_3CHC(CH_3)OH \rightarrow CH_3CH(C)CH_2CHOH$	IRC		a	3	83.32	87.77	N/A	693	400
41	$CH_3CHC(CH_3)OH \rightarrow CH_2CCHCH_3 + OH_2$			a	3	80.87	81.77	-0.90	1836	1620
42	$CH_3CHC(CH_3)OH + OH_2 \rightarrow CH_3CH_2C(CH_3)(OH)OH$			a	3	49.59	49.65	-0.05	1823	1793
43	$CH_3CHC(CH_3)OH \rightarrow CH_3CH_2C(CH_3)O$			a, d	2	53.70	53.70	0.00	2141	2141
44	$CH_3CHC(CH_3)OH + OH_2 \rightarrow CH_3CH(OH)CH(CH_3)OH$			a	3	66.65	66.50	0.15	1999	2060
45	$CH_3CHC(CH_3)OH + OH_2 \rightarrow CH_3CHC(CH_3)OH + OH_2$			a	4	40.82	41.58	-0.76	2009	2117
46	$CH_3CHC(CH_3)OH + OH_2 \rightarrow CH_3CHC(CH_3)OH + OH_2$			a	4	68.36	68.35	0.00	1270	1270
47	$CH_3CHC(CH_3)OH + OH_2 \rightarrow CH_3CH_2C(CH_2)OH + OH_2$			a	4	49.67	48.80	0.87	2075	2011
48	$CH_3CHC(CH_3)OH \rightarrow CH_2CHCH(CH_3)OH$			a, d	2	83.43	83.43	0.00	1749	1750

Continued on next page

Table S1: Overview of errors, amount of changing bonds, barrier energies and imaginary frequencies for all reactions for all optimised TSs and reference TSs. (Continued)

ID.	Reaction	Fail	Ref fail	Note	Bonds	$E^{\ddagger}_{\text{opt.}}$ kJ/mol	$E^{\ddagger}_{\text{ref.}}$ kJ/mol	$\Delta E^{\ddagger}$ kJ/mol	ω_0 cm^{-1}	$\omega_{0,\text{ref.}}$ cm^{-1}
49	$CH_3CHC(CH_3)OH + OH_2 \rightarrow CH_3CH(O)CH(O)CH_3 + OH_2$	IRC	IRC	a	5	82.79	71.22	N/A	617	549
50	$CH_3CHC(CH_3)OH + OH_2 \rightarrow CH_3CH(OH)C(O)CH_3 + H_2$		IRC	a	4	99.00	100.20	-1.20	2132	1458
51	$CH_2O + CH_3OH \rightarrow OHCH_2OCH_3$			a	3	67.94	67.94	0.00	648	649
52	$CH_2O + CH_3OH \rightarrow CH_3OCH_2OH$			a	3	34.13	34.12	0.00	1662	1662
53	$CH_2O + CH_3OH \rightarrow CH_3OH + CH_2O$			a	4	28.29	28.29	0.00	1433	1434
54	$CH_2O + CH_3OH \rightarrow CH_3OH^i + CH_2O$			a	4	47.94	47.94	0.00	1798	1797
55	$CH_2O + CH_3OH \rightarrow CH_3OH + CO{+}H_2$			a	5	66.11	66.10	0.01	1894	1887
56	$CH_2O + CH_3OH \rightarrow OHCH_2CH_2OH$			a	3	81.33	81.32	0.01	2031	2032
57	$CH_3CHNCH_3 \rightarrow CH_2CHNHCH_3$			a	2	65.64	65.64	0.00	1974	1973
58	$CH_3CHNCH_3 \rightarrow CH_3CH(N)CH_2NH$		IRC	a	3	82.78	71.39	11.39	583	1647
59	$CH_3CHNCH_3 \rightarrow CH_3CNHCH_3$			a	2	91.75	91.75	0.00	1448	1449
60	$CH_3CHNCH_3 \rightarrow CH_3CHNHCH_2$			a	2	71.39	71.39	0.00	1665	1665
61	$CH_3PHCH_3 + CH_2CH_2 \rightarrow CH_3CH_2PH(CH_3)CH_2$			a	3	46.48	46.48	-0.01	1357	1357
62	$CH_3CHCH_2 \rightarrow CH_2CHCH_3$			a	2	82.29	82.15	0.14	1863	1293
63	$CH_3CHCH_2 \rightarrow CH_2(C)CH_2CH_2$	IRC	IRC	a	5	82.56	82.56	N/A	145	117
64	$H_2 + SiH_2 \rightarrow SiH_4$	IRC		a	3	0.71	-2.59	N/A	105	1134

Continued on next page

Table S1: Overview of errors, amount of changing bonds, barrier energies and imaginary frequencies for all reactions for all optimised TSs and reference TSs. (Continued)

ID.	Reaction	Fail	Ref fail	Note	Bonds	$E^{\ddagger}_{\text{opt.}}$ kJ/mol	$E^{\ddagger}_{\text{ref.}}$ kJ/mol	$\Delta E^{\ddagger}$ kJ/mol	ω_0 cm^{-1}	$\omega_{0,\text{ref.}}$ cm^{-1}
65	$S(O_2)Cl_2 \rightarrow OSO + Cl_2$	FRQ		a, d	3	-0.31	57.39	N/A	-188	526
66	$CH_3CHNCH_3 \rightarrow CH_4 + CNCH_3$		IRC	a	3	115.15	123.39	-8.24	649	1369
67	$CH_3C(CH_3)CH_2 + OH_2 \rightarrow CH_3C(CH_3)(CH_3)OH$			a	3	50.11	50.10	0.01	1937	1936
68	$CH(CH(CH_3)CH(CH_3)CH)C \rightarrow CH_3CHCHCHCHCH_3$			a	1	31.39	31.37	0.02	535	537
69	$CH_2CHCHCH_2 + CH_2CH_2 \rightarrow CHCHCH_2CH_2CH_2CH_2$			a	2	15.91	15.91	0.00	522	522
70	$CH_2CHCHCH_2 + CH_2CH_2 \rightarrow CHCH(CH_2)CH_2CH_2CH_2$			a	3	56.23	56.23	0.00	728	728
71	$CH_2CHCHCH_2 + CH_2CH_2 \rightarrow CH_3CH_2CHCHCHCH_2$	IRC		a	3	15.91	91.04	N/A	520	1856
72	$CH_2CHCHCH_2 + CH_2CH_2 \rightarrow CH_2CHCH_2CHCHCH_3$			a	3	38.11	38.12	-0.01	431	431
73	$CH_2CHCHCH_2 + CH_2CH_2 \rightarrow CH_2CHCH(CH_3)CHCH_2$	IRC		a	3	-1.99	67.28	N/A	16	711
74	$CH_2CHCHCH_2 + CH_2CH_2 \rightarrow CH_2CHCH(CH_2)CHCH_3$	IRC	IRC	a	4	64.89	75.30	N/A	1291	85
75	$CH_2CHCHCH_2 + CH_2CH_2 \rightarrow CH_2CHCH(CH_2)CHCH_3$[i]		IRC	a	4	80.58	75.04	5.54	1281	53
76	$CH_2CHCHCH_2 + CH_2CH_2 \rightarrow CH_2CHCH_2CHCH_2CH_2$	IRC	IRC	a, e	4	81.24	81.23	N/A	1383	1382
77	$CH_2CHCHCH_2 + CH_2CH_2 \rightarrow CH_3CH_2CH_2CHCCH_2$	IRC	IRC	a, e, f	3	36.19	36.19	N/A	969	973
78	$CH_2CHCHCH_2 + CH_2CH_2 \rightarrow CH_2CH_2 + CH_2CHCHCH_2$	IRC		a	4	92.11	124.19	N/A	1546	1762
79	$CH_2CHCHCH_2 + CH_2CH_2 \rightarrow CH_2CCHCH_3 + CH_2CH_2$	IRC	IRC	a, e, f	4	64.70	64.08	N/A	1690	1659
80	$CH_2CHCHCH_2 \rightarrow CH(CH_2CH_2CH)C$			a	1	44.00	44.00	0.00	738	738

Continued on next page

Table S1: Overview of errors, amount of changing bonds, barrier energies and imaginary frequencies for all reactions for all optimised TSs and reference TSs. (Continued)

ID.	Reaction	Fail	Ref fail	Note	Bonds	$E^{\ddagger}_{\text{opt.}}$ kJ/mol	$E^{\ddagger}_{\text{ref.}}$ kJ/mol	$\Delta E^{\ddagger}$ kJ/mol	ω_0 cm^{-1}	$\omega_{0,\text{ref.}}$ cm^{-1}
81	$CH_2CHCHCH_2 + CH_2CH_2 \rightarrow CH_3CH_2CH_2CHCCH_2$	IRC	IRC	a, e, f	3	36.20	36.20	N/A	969	969
82	$CH_3P(CH_3)CH_3 + CH_2(CH_2O)O \rightarrow P(CH_3)(CH_3)CH_2CH_2O$			a	3	48.47	48.48	-0.01	428	428
83	$CH_3CHCHCH_2OH + OSCl_2 \rightarrow CH_3C(OH)CHCH_2OH + ClSCl$			a	4	79.01	80.98	-1.97	890	914
84	$CH_3CHCHCH_2OH + OSCl_2 \rightarrow CH_3CHCHCH_2OS(O)Cl + ClH$			a	6	52.45	52.45	0.00	1623	1624
85	$CH_3CHCHCH_2OH + OSCl_2 \rightarrow CH_3CHCHCH_2OS(O)Cl^{i} + ClH^{i}$	IRC	IRC	a	4	19.58	35.41	N/A	100	385
86	$CH_3CHCHCH_2OH + OSCl_2 \rightarrow CH_3CH(O)CHCH_2OH + ClSCl$			a	3	54.87	52.09	2.78	529	514
87	$CH_3CHCHCH_2OH + OSCl_2 \rightarrow CH_3C(O)CHCH_2OH + ClSCl$			a	3	51.63	48.81	2.82	459	538
90	$C_5H_6OCH_2CHCH_2 \rightarrow C_5H_6CH_2CH(O)CH_2O$	IRC	IRC	a	5	34.39	87.25	N/A	413	447
91	$C_5H_6OCH_2CHCH_2 \rightarrow C_5H_6CH_2CH(O)CH_2O^{i}$			a	3	84.23	84.23	0.01	777	777
92	$C_5H_6OCH_2CHCH_2 \rightarrow C_5H_6OCH(CH_2CH_2)C$			a	3	75.12	75.12	0.00	556	557
93	$CH_3C(O)OOH \rightarrow OHOC(CH_3)O$	FRQ		a, d	2	58.82	55.06	N/A	609	420
94	$CH_3C(O)OOH + CH_3CHCHCH_3 \rightarrow CH_3CHC(CH_3)C(CH_3)(OH)OOH$			a	3	73.28	73.25	0.03	1901	1764
95	$CH_3C(O)OOH + CH_3CHCHCH_3 \rightarrow CH_3CHC(CH_3)OCH(CH_3)OOH$		IRC	a	3	96.70	98.40	-1.69	1447	1398
96	$CH_3C(O)OOH + CH_3CHCHCH_3 \rightarrow CH_3C(OH)O + CH_3CH(CH(CH_3)O)O$			a	5	16.79	17.23	-0.45	436	444
97	taxadiene carbocation $\rightarrow$ taxadiene carbocation^{i}	FRQ	IRC	a	2	8.93	1.24	N/A	705	39
98	taxadiene carbocation $\rightarrow$ taxadiene carbocation^{ii}		IRC	a	2	1.11	1.25	-0.14	429	50

Continued on next page

Table S1: Overview of errors, amount of changing bonds, barrier energies and imaginary frequencies for all reactions for all optimised TSs and reference TSs. (Continued)

ID.	Reaction	Fail	Ref fail	Note	Bonds	$E^{\ddagger}_{\text{opt.}}$ kJ/mol	$E^{\ddagger}_{\text{ref.}}$ kJ/mol	$\Delta E^{\ddagger}$ kJ/mol	ω_0 cm^{-1}	$\omega_{0,\text{ref.}}$ cm^{-1}
99	taxadiene carbocation → taxadiene carbocation[iii]		IRC	a	2	6.59	-0.57	7.16	273	141
100	$CH_2CH_2 + N_2O \rightarrow CH_2(NNOCH_2)C$			b	2	25.72	25.72	0.00	477	478
101	$CH_2CHCHCHCH_3 \rightarrow CH_3CHCHCHCH_2$			b	2	32.79	32.79	0.00	1537	1536
102	$CHN \rightarrow CNH$			b	2	47.86	47.86	0.00	1123	1123
103	$CH_2CHCH_2CH_2CHCH_2 \rightarrow CH_2CHCH_2CH_2CHCH_2$			b	2	32.27	32.27	0.00	492	492
104	$CHCHCHCH_2CH \rightarrow CHCHCHCH_2CH$[i]			b	2	26.53	26.53	0.00	1188	1187
105	$2\ CHCHCHCH_2CH \rightarrow CH(CHCH(CH(CH_2)CH(CHCHCHCH_2)))$			b	2	14.48	14.48	0.01	497	497
106	$CH_2CH_2 + CHCHCHCH_2CH \rightarrow CH(CH(CH_2CH(CH)CH_2CH_2))$			b	2	16.83	16.82	0.00	500	500
107	$CH_2CH_2 + CF_2 \rightarrow CH_2CH_2CF_2$			b	2	8.70	8.70	0.00	384	384
108	$CH_2CHCH_2CH_2CH_3 \rightarrow CH_2CH_2 + CH_3CHCH_2$			b	3	55.67	55.67	0.00	1164	1166
109	$BrMg(C_5H_6)_2OC(C_5H_6)_2 \rightarrow BrMgOC(C_5H_6)_3$	FRQ	FRQ	b	3	17.98	17.97	N/A	216	215
110	$H_2 + CO \rightarrow CH_2O$	IRC		b	3	0.25	73.99	N/A	90	1881
111	$CH_2CH_2 + FH \rightarrow CH_3CH_2F$			b	3	43.85	43.85	-0.01	1820	1820
112	$CH_3C(O)OCH_2CH_3 + 2\ OH_2 \rightarrow CH_3C(OH)O + CH_3CH_2OH + OH_2$	IRC		b	6	30.96	35.12	N/A	1241	1255
113	$CH_2O{+}H_2 \rightarrow CH_3OH$		IRC	b	3	67.79	76.03	-8.24	2231	1393
115	$CH_2CHCH_2OCHCH_2 \rightarrow CH_2CHCH_2CH_2CHO$			b	2	64.04	63.30	0.74	480	401

Continued on next page

Table S1: Overview of errors, amount of changing bonds, barrier energies and imaginary frequencies for all reactions for all optimised TSs and reference TSs. (Continued)

ID.	Reaction	Fail	Ref fail	Note	Bonds	$E^{\ddagger}_{\mathrm{opt.}}$ kJ/mol	$E^{\ddagger}_{\mathrm{ref.}}$ kJ/mol	$\Delta E^{\ddagger}$ kJ/mol	ω_0 cm^{-1}	$\omega_{0,\mathrm{ref.}}$ cm^{-1}
116	$CH_3C(CH_3)(CH_3)F + Cl \rightarrow CH_3C(CH_3)(CH_3)Cl + F$			b	2	45.59	45.60	-0.01	398	398
117	$CH(CHCH_2S(O)(O)CH_2)C \rightarrow CH_2CHCHCH_2 + OSO$			b	2	19.07	19.07	0.00	334	334
118	$CH_2CHCH_2OCHCH_2 \rightarrow CH_2CHCH_2CH_2CHO$		IRC	b	2	28.02	31.96	-3.93	492	458
119	$BH_3NH_3 + BH_2NH_2 \rightarrow BH_3NH_3^{i} + BH_2NH_2^{iv}$	IRC	IRC	b	4	0.96	2.47	N/A	158	858
120	$BH_3NH_3 + BH_2NH_2 \rightarrow BH_3NH_3^{ii} + BH_2NH_2^{v}$	IRC	IRC	b	4	42.83	52.71	N/A	1381	1469
121	$BH_3NH_3 + BH_2NH_2 \rightarrow BH_3NH_3^{iii} + BH_2NH_2^{vi}$		IRC	a, c	4	37.69	39.31	-1.62	583	919
122	$CH_2CHOH + CH_2O \rightarrow OHCH_2CH_2CHO$			a c	3	11.98	11.98	0.00	793	793
123	$CH_2CHCHCH_2 \rightarrow CH(CH_2CH_2CH)C$			b, c	1	45.66	45.66	0.00	729	729
